# Finding mechanisms from metabolic signatures of disease

**DOI:** 10.1186/1753-6561-6-S3-O19

**Published:** 2012-06-01

**Authors:** Christopher B Newgard

**Affiliations:** 1 Sarah W Stedman Nutrition and Metabolism Center & Department of Pharmacology & Cancer Biology, Duke University Medical Center, Durham, NC, USA

## 

We seek to apply comprehensive metabolic analysis tools (sometimes called “metabolomics”) for understanding of mechanisms underlying chronic human diseases and conditions such as diabetes, obesity, and cardiovascular disease. Current approaches include analysis of metabolic flux by ^13^C NMR-based mass isotopomer analysis (in collaboration with Drs. Shawn Burgess and A. Dean Sherry and associates, Dallas, TX) and metabolic profiling of important groups of metabolic intermediates by both “targeted” and “unbiased” mass spectrometry (in collaboration with Drs. James Bain, Robert Stevens, Olga llkayeva, Brett Wenner, Michael Muehlbauer, Mark Butler, and David Millington at Duke). These tools have also been used to define mechanisms underlying development of peripheral insulin resistance in animals and humans. For example, we have recently identified perturbations of branched chain amino acid (BCAA) catabolism in multiple cohorts of insulin resistant humans compared to normally insulin sensitive controls and have translated these findings to rodent models to demonstrate a contribution of BCAA to development of insulin resistance that is independent of body weight. In collaboration with Dr. Alan Attie at the University of Wisconsin, we have integrated transcriptomic and metabolomic analysis in mouse models to identify new pathways that control hepatic gluconeogenesis and PEPCK expression. Finally, with Svati Shah and Bill Kraus at Duke, we have identified novel metabolic signatures of imminent cardiovascular events, and are integrating genomic and metabolomics analyses in large cohorts of human subjects with a high incidence of coronary artery disease to identify pathways involved in metabolic variability and risk of cardiovascular disease. These examples will serve to illustrate the potential of comprehensive metabolic profiling methods for providing insights into metabolic disease mechanisms.

